# Domains of Everyday Creativity and Personal Values

**DOI:** 10.3389/fpsyg.2018.02681

**Published:** 2019-01-14

**Authors:** Nadezdha Lebedeva, Shalom H. Schwartz, Fons J. R. Van De Vijver, Jonathan Plucker, Ekaterina Bushina

**Affiliations:** ^1^Department of Psychology, National Research University Higher School of Economics, Moscow, Russia; ^2^Department of Psychology, Hebrew University of Jerusalem, Jerusalem, Israel; ^3^Department of Culture Studies, Tilburg University, Tilburg, Netherlands; ^4^School of Education, Johns Hopkins University, Baltimore, MA, United States

**Keywords:** global creativity, domains of creativity, values, cultural regions, Russia, North Caucasus, hybrid model of creativity

## Abstract

We examined the applicability of the hybrid model of creativity, which specifies distinct domains that all express an underlying general creativity factor, in data from representative samples from Central Russia and the North Caucasus (*N* = 2,046). Using multigroup confirmatory analysis, Study 1 supported the invariance of a model with the six unifactorial domains (i.e., crafts, visual arts, performance, theater, products for work, and machine graphics) at the first level and a general creativity factor at the second level. Study 2 examined socio-demographic characteristics and 19 basic values that might be associated with creative activity. The more modern Central Russian region scored higher on global creativity and on all 6 domains. Of the 4 higher order values in the Schwartz model, Openness to Change values correlated positively and Conservation values correlated negatively with global creativity and with creativity in most domains. Variation across domains in the specific values that predicted creativity revealed that creativity in each domain had some unique motivators. We draw on culture and social structure to explain differences between regions in the value motivators of creativity.

Creativity is vital to society. It facilitates and enhances problem solving, enabling progress across economic, scientific, social, and artistic domains (Runco, 2004; Hennessey and Amabile, 2010). Creativity's close links to such beneficial characteristics of performance as change, innovation, reform, and progress explain the proliferation of studies aimed at identifying its social, cultural, personal, and other determinants. This study seeks to identify distinguishable domains of everyday creative behavior and to examine motivational and cultural variables that might account for individual differences in this behavior. The present study adds to the literature in two ways. First, we extend current approaches to creativity by specifying a hybrid, hierarchical model that encompasses both a general and a domain-specific aspect of creativity. We test the applicability of the model in a more traditional and a more modern part of the Russian Federation. Second, we examine associations of creativity with basic values and with various sociocultural characteristics. As argued below, such associations can be expected, yet these have infrequently been studied.

The meaning of “creativity” varies across the social sciences. Personal creativity is emphasized in education, entrepreneurship in business, problem solving in mathematics, and aesthetic products in art (Reid and Petocz, 2004). The creativity construct refers to four levels of psychological reality and their fields of research: (1) the individual who is the subject of creativity, (2) the cognitive processes involved in producing creative ideas, (3) the environment in which creative acts occur and which influences them, and (4) the product or outcome of creative activity (Goldenberg et al., 1999; Runco, 2004).

Most scholars agree that creativity is a quality of individuals or a type of process that provides suitable new, atypical solutions to problems (Mayer, 1999). They also agree that creativity entails producing novel, useful outcomes that are recognized as such by experts in a relevant field (Amabile, 1983, 1996; Brown, 1989; Mayer, 1999; Hennessey and Amabile, 2010). The current research adopts the following definition of creativity from the Plucker et al. (2004) review of conceptualizations of creativity:

Creativity is the interaction among aptitude, process, and environment by which an individual or group produces a perceptible product that is both novel and useful as defined within a social context (p. 90).

## Creativity or Creativities?

The importance of context raises the question of specificity vs. universality of creativity across domains. If creativity is judged differently in different fields, does that imply different types of creativity specific to those fields? Or is creativity a general phenomenon, common across domains? In other words, is it better to speak of one creativity or many creativities? This has been one of most enduring, polarized debates about creativity, with strong support mustered for both positions (e.g., Runco, 1987; Brown, 1989; Simonton, 2012).

Historically, the domain-general perspective dominated. For example, early tests of creativity as a trait implied its universality (Torrance, 1974), echoing Spearman's model of general intelligence (*g*). Guilford (1968) proposed four characteristics of general creativity: productivity (fluency), flexibility, originality, and complexity (elaboration). Presumably, these were all equally useful across domains (Plucker and Makel, 2010).

However, the dominant perspective has shifted to domain specificity over the past 20 years. This perspective views creativity as domain-specific, independent sets of features necessary to achieve a high level of innovative performance (Baer, 1998; Kaufman and Baer, 2004). Csikszentmihalyi's (1988) systems model of creativity, which focuses on the interaction of the individual, subject of creativity, scope and field of creative activity, strongly implies a domain specific approach. Although the style of activity is similar across contexts, the abilities and skills required for high-level creativity differ greatly from sphere to sphere because the domain and field contexts directly impact their formation. In a particularly strong statement favoring domain specificity, Baer (1994) argued that the traits, characteristics, and skills required for a high level of creativity are so specific to each domain that they cannot be transferred to others and can determine creativity only in a complementary field.

We adopt a third, hybrid point of view that balances these two poles. It considers creativity as a partially universal ability with both domain and task general and specific components (e.g., Amabile, 1996; Plucker, 2005). The degree of specificity or generality depends both on the social context and on development from childhood into adulthood. For example, Amabile (1983) proposed a three-component model of creativity: (a) skills required for a particular area (e.g., knowledge and talents), (b) general skills and abilities associated with creativity (e.g., cognitive style and divergent thinking), and (c) motivation to solve particular problems.

Baer and Kaufman (2005) proposed another hybrid model of creativity: (a) basic features and capabilities needed for all creativity (e.g., intelligence, motivation, and suitable environmental factors); (b) both general (e.g., empathy and communication) and specific (e.g., mathematical/scientific) abilities; (c) specific creative fields (e.g., music, arts and crafts, and poetry); and (d) even more specific creative activities (e.g., writing novels and performing jazz) that typically correspond to professions.

In a third hybrid model, Plucker and Beghetto (2004) argued that creativity is a developmental construct that exhibits both domain-specific and domain-general characteristics. They attributed the domain-specific nature of creativity to the complex interplay between individuals' interests and motivations for specific, potentially creative tasks on the one hand and their age and experience on the other. As both interest/motivation and age/experience increase, products appear to be more domain specific. This is because increasing personal and professional responsibilities limit people's time to work creatively across multiple, related domains. However, there is potential transfer of general creative abilities across domains and tasks. This latter model, which combines the general and domain specific models, underlies the current study. Study 1 tests the applicability of this model in samples of adults from culturally various distinct regions of the Russian Federation.

## Sociocultural Approaches to the Study of Creativity

Most theorizing and research on creativity to date has been conducted in the United States and Western Europe. But views of what constitutes creativity and of how important various types of creativity are across cultures may differ. Yet empirical research on creativity has rarely addressed sociocultural variables. Culture may influence the generality of creativity, its specific domains, and factors that promote different types of creativity (Glăveanu, 2010, 2014). Although creativity and artistic expression are universal phenomena, implicit theories of what constitutes creativity differ across cultures (Rudowicz, 2003). Western societies (e.g., USA and Europe) emphasize novelty, originality, and self-expression; Eastern societies (e.g., China, Japan, and Korea) view interpretations of existing traditions as creative solutions (Rudowicz, 2003; Pang and Plucker, 2013).

The current research studied creative behavior of representative samples from two federal regions of Russia, Central Russia and the North Caucasus. These regions differ substantially in cultural and socio-demographic characteristics. Central Russia is one of the most urbanized, modern, and economically developed districts of the Russian Federation, with the largest population, consisting of about 90% ethnic Russians who are largely Russian Orthodox. The North Caucasus is over 50% rural, agricultural, culturally traditional, economically underdeveloped, and has the smallest population in the Russian Federation. It includes many ethnic groups, most Muslim, and is the only federal district in which ethnic Russians are in the minority.

We sought to identify distinguishable domains of creative behavior and to examine motivational and socio-demographic variables that might account for individual differences in the frequency of creativity. We examined whether the same domains of creativity were distinguishable across regions. We were particularly interested in whether the motivations for creativity might differ across domains. The basic values of individuals served as our measure of their motivations. For the domains of creative behavior identified, we asked whether the effects of values, gender, religion, religiosity, and education on levels of creativity differed across regions. Thus, we considered culture as a moderator variable.

This article presents two sequential studies. The first study is more conceptual by examining whether it is possible to distinguish distinct domains of creativity in the two cultural contexts and whether these domains express a general, underlying creativity factor, in line with hybrid views on creativity. More specifically, we tested the cross-group invariance of a second-order factor model in which the creativity domains are the factors (with items as their markers) at the lowest level and the general creativity factor (with the first-order factors as markers) is at the second level. Study 2 examines the value (motivational) and socio-demographic variables related to creativity in the two cultural contexts (hypotheses are explained in the description of Study 2).

## Study 1. Domain Specificity and Generality in Creativity

### Method

#### Participants and Procedure

A survey research organization conducted face-to-face interviews with stratified random samples of adults aged 20–60 in the Central and North Caucasus federal districts of Russia in June-August 2012. The organization has its own samples and has its own forms to obtain informed consent. Participants gave written informed consent. This procedure is in line with Russian regulations; as per university and national Russian regulations, no ethics clearance is required for this type of survey research (if it does not include medical data).

The Central district sample included 1,020 respondents [52% female, mean age 38.8 years (*SD* = 12.3), 29.9% with bachelor's degree, 94.6% Russian Orthodox]. The North Caucasus district sample included 1,026 respondents [52% female, mean age 36.6 (*SD* = 12.4), 29.7% with bachelor's degree, 33.0% Russian Orthodox, 64.6 % Muslim].

#### Creativity Instrument

We adapted the 25-item Creative Behavior Inventory (CBI) from Dollinger (2003). Each item describes a type of everyday creative behavior that respondents may have performed. The Dollinger (2003) items refer mainly to behaviors from the arts and crafts, which corresponds to our interest in assessing creativity in our sample. Supporting the validity of the CBI are correlations with many other markers of creativity (e.g., Dollinger et al., 2005; Silvia et al., 2012). We expected these correlations that support the validity of the measure also to hold in Russia. A one-factor model derived by exploratory factor analysis fit the items well (Silvia et al., 2012). This combination of a global factor that leaves room for domain-specific aspects is in line with our expectation of the structure of creativity in our study, as explained above.

We translated the CBI into Russian using double reverse translation with native English and Russian speakers. Of the original 28 items, we modified four items and combined 11 original, mostly crafts, items into three items. We added nine new items intended to tap other forms of creativity including creative activity within organizations and work with graphics. The Appendix presents the revised questionnaire, distinguishing the new and modified items. Respondents indicated how frequently, if at all, they had ever performed each activity on a 4-point scale: 1—Never did this, 2—Did this once or twice, 3—Did this 3–5 times, 4—Did this more than 5 times.

Although previous research with the Dollinger (2003) CBI had supported a one-factor structure, we expected to find, in addition, domain-specific, lower-order factors. Our addition of items that measured domains other than arts and crafts was likely to yield such specific factors. Examination of the content of our modified CBI suggested six potentially distinguishable domains of creative behavior. We labeled these domains: visual arts, crafts, work products, public performance, theater, and machine graphic. We expected these domains, in turn, to load on a higher-order general creativity factor.

In preliminary analyses (exploratory factor analysis) we found that five items showed either strong secondary loadings or did not show a strong loading on any factor. These were items 7, 10, 11, 12, and 16 (see the Appendix). These items were excluded from the remaining analyses, leaving a creativity instrument of 20 items.

### Results

A multigroup confirmatory factor analysis on the data from the two cultural samples assessed our model and tested whether the factor structure was similar across the two regions (*N* = 2,046). In preliminary analyses we found that some correlated error terms between rather similar items were needed to achieve a good fit (item 1 and 2, item 13 and 14; item 22 and 23). We tested a higher-order model of creativity with the six domains at the first-level model and a general creativity factor at the second level. This model acknowledges that creative behaviors show some domain specificity, but a general creativity factor underlies all these behaviors. Invariance of the model followed a multistep procedure (cf. Chen et al., 2005; Chen, 2007; Rudnev et al., 2018). The procedure tests the invariance of the level-1 factors (associations of items to domains), followed by the testing of the invariance of the level-2 general creativity factor.

The fit results are presented in Table 1. The invariance test of the configural invariance model (testing the same patterning of loadings at level 1 and level 2) showed fairly positive results. The χ^2^ statistic was highly significant, but the χ^2^/*df* was 2.08, which is acceptable. The other fit statistics also showed rather positive results. The metric invariance level-1 model (testing whether factor loadings were invariant across the regions) was less strongly supported, with a rather strong increase of the χ^2^ statistic, a decrease of the CFI value of just over 0.01, a larger value of the BIC (adjusted for sample size) and SRMR values. An inspection of modification indices (not further reported here) did not suggest specific model changes in the model. Interestingly, the test of the level-1 scalar invariance model (testing the presence of differential item functioning) yielded fairly favorable results (see Table 1). All in all, we concluded that the invariance of the level-1 structure is supported and that the split in six creativity domains is supported by the data.

**Table 1:** Invariance analysis of the second-order structure of creativity (Level 1: Associations of items and domains; Level 2: Association of general creativity and domains).

**Model**	**χ^2^/*df***	**CFI**	**ΔCFI**	**RMSEA**	**BIC**	**SRMR**
Level 1: Configural invariance^a^	664.51/320	0.908		0.032	60259	0.056
Level 1: Metric invariance^a^	718.92/334	0.897	0.011	0.034	60402	0.067
Level 1: Scalar invariance^a^	766.41/348	0.888	0.009	0.034	60423	0.066
Level 2: Metric Invariance^b^	795.83/353	0.882	0.006	0.035	60508	0.074
Level 2: Scalar Invariance^b^	818.34/358	0.877	0.005	0.035	60536	0.074

a *Assuming configural invariance at level 2*.

b *Assuming scalar invariance at level 1*.

These results provided the support required for testing the invariance of the general factor at level 2. Both the metric and scalar invariance tests at level 2 showed favorable results, thereby supporting the invariance of the general creativity factor at level 2. The factor loadings of the scalar invariance model are given in Table 2. Combining the results of the invariance tests at both levels, it can be concluded that the layered conceptualization of creativity, as postulated in the hybrid model, is supported in our data.

**Table 2:** Domain reliabilities and factor loadings.

**Domain**	**Loading on general factor^a^**	**Item #**	**Brief item content**	**Loading^a^**
Crafts α = 0.72, 0.58	1.43	5	Made a decorative craft (from metal, plastic, glass, leather, ceramics, wood, beads, jewelry)	1.00^c^
		6	Made costumes, designed and made clothes, embroidered	0.93
		21	Prepared an original floral arrangement or garden design	0.69
Visual arts α = 0.71, 0.66^b^	0.80	1 2 3	Painted an original picture Made a sculpture Made sketches, …paintings….	1.00^c^ 0.51 1.51
Performance α = 0.75, 0.76	0.94	8	Performed on a musical instrument in a concert or on the street ….	1.00^c^
		9	Performed as singer alone, in ensemble or chorus on stage or street	1.24
		13	Performed as dancer alone, in ensemble on stage or street	0.63
Theater α = 0.63, 0.76	0.50	14 15	Created or choreographed a dance for performance Put on a puppet show	1.00^c^ 0.96
		17	Directed a play or other theatrical performance	1.05
		18	Acted in a play, other theater performance or movie	1.55
Products for work α = 0.72, 0.69	1.00^c^	22 23	Developed a new procedure, rule, work arrangement that was adopted Developed a new product (machine, hardware/software, etc.)	1.00^c^ 0.55
		24	Made an architectural plan for a building, house, flat, landscape	0.65
		25	Made original posters, placards for work—for public meetings	1.16
Machine graphics α = 0.45, 0.66	0.96	4 19	Made a picture, collage, web-site or other with computer graphics Drew or made cartoons on a computer to show to other people	1.00^c^ 0.45
		20	Made a movie to show to other people	0.43

a *All loadings are significant (p < 0.001)*.

b *The first number refers to the reliability in the sample from Central Russia, the second to the reliability in the sample from the North Caucasus. The internal consistency of the global factor was 0.91 in both samples*.

c *Loading fixed at a value of 1*.

### Discussion

Study 1 tested a hybrid model in which creativity comprises both general and domain-specific components. We tested this model in two culturally very different Russian regions (Central Russia and the North Caucasus). The data supported the hybrid model in both regions. Thus, though the hybrid model was developed mainly based on Anglo-Saxon data, it can be extended to the Russian context.

## Study 2. Values and Creativity

Study 2 examined associations between background characteristics and creativity in the cultural contexts of our study. In addition, we addressed possible individual characteristics that predict creativity (both the general factor and each of the six domains of creativity). In particular, we were interested in the motivations that propel creativity. We used individuals' basic personal value priorities to measure motivations. These associations have infrequently been addressed before, although there is widespread appreciation of the links between individual and societal characteristics and creativity. Our study of the role of background characteristics was largely exploratory. However, links between values and creativity were studied within a comprehensive theory of human values. This permitted the formulation of several hypotheses, as explained below in more detail.

**Figure 1:**
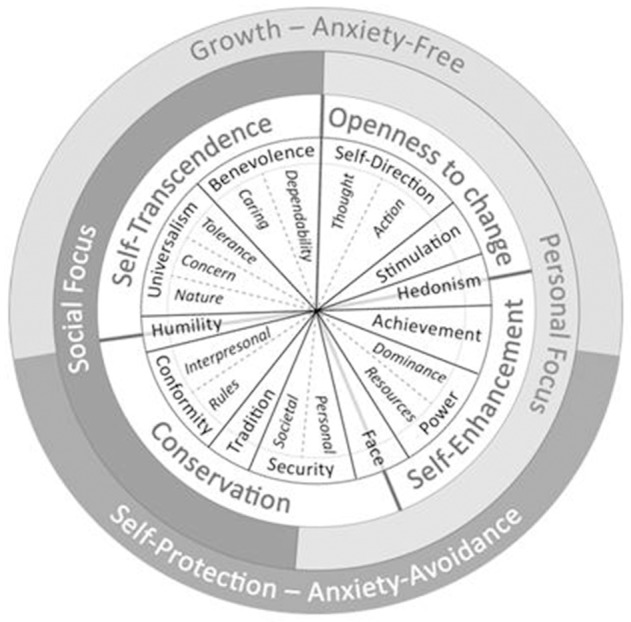
The motivational circle of values with 19 values, the ten original values and the dynamic bases that organize and give coherence to value systems (adapted from Schwartz et al., 2012).

Schwartz (1992) identified 10 distinct values that form a circular structure based on their motivational compatibility or opposition. Schwartz et al. (2012) discriminated 19 distinct values arrayed in the same motivational circle. These values are recognized across cultures and motivate and predict attitudes and behaviors (Schwartz et al., 2012, 2017). Figure 1 presents the value circle with the 19 values, the 10 original values, and the dynamic bases that organize values in the motivational circle. Table 3 presents the motivational goals of the 19 values whose associations with creative behaviors we examined.

**Table 3:** The 19 basic values in the refined theory, each defined in terms of its motivational goal.

**Value**	**Motivational goal**
Self-direction—thought	Freedom to cultivate one's own ideas and abilities
Self-direction—action	Freedom to determine one's own actions
Stimulation	Excitement, novelty, and change
Hedonism	Pleasure and sensuous gratification
Achievement	Success according to social standards
Power—dominance	Power through exercising control over people
Power—resources	Power through control of material and social resources
Face	Security and power through maintaining one's public image and avoiding humiliation
Security—personal	Safety in one's immediate environment
Security—societal	Safety and stability in the wider society
Tradition	Maintaining and preserving cultural, family or religious traditions
Conformity—rules	Compliance with rules, laws, and formal obligations
Conformity—interpersonal	Avoidance of upsetting or harming other people
Humility	Recognizing one's insignificance in the larger scheme of things
Benevolence—dependability	Being a reliable and trustworthy member of the in-group
Benevolence—caring	Devotion to the welfare of in-group members
Universalism—concern	Commitment to equality, justice and protection for all people
Universalism—nature	Preservation of the natural environment
Universalism—tolerance	Acceptance and understanding of those different from oneself.

Previous research has demonstrated relations of several values to the general factor of creativity (e.g., Dollinger et al., 2007). Studies have most consistently found that the values of self-direction, universalism, and stimulation relate positively to global creativity whereas the values of tradition, conformity, and security relate negatively. Kasof et al. (2007) observed that correlations of creativity are positive and strongest with self-direction values and become less positive and more negative as one goes around the value circle in both directions toward tradition values (see Figure 1). Power related negatively to global creativity in one study (Dollinger et al., 2007).

Based on these findings and the motivational goals of the 19 basic values studied here, we generated hypotheses regarding the associations between values and creativity. Especially relevant was the understanding that creativity typically entails performing activity with novel outcomes. Consequently, we expected the higher order Openness to Change value, whose pursuit involves self-expansion in ever-changing directions, to relate positively to creativity. In contrast, we expected the higher order Conservation value, whose pursuit involves protecting the self by maintaining a stable social and physical environment, to relate negatively to creativity. We expected no association for the higher order Self-Enhancement value. We expected only a weak positive association for the higher order Self-Transcendence value because of expected associations of Universalism-Nature with creativity in four domains and of the two Benevolence values with creativity in two domains (see below).

Following are the hypotheses for ten specific values. In parentheses, we list the motivational goals that explain their expected associations with global creativity and with creativity in most domains. Values that motivate creativity and should relate positively to it: Self-direction Thought and Self-direction Action (pursuit of independence of thought and action), Stimulation (pursuit of novelty and change), Hedonism (pursuit of pleasurable arousal), and Universalism-Nature (an interest in the aesthetic). Values that inhibit creativity and should relate negatively to it are: Tradition (preserving traditional and accepted modes of thought and action), Conformity-Rules and Conformity-Interpersonal (avoiding violation of norms, conventions, and interpersonal expectations and eliciting formal or informal sanctions), Humility (avoiding immodesty or standing out), and Security-Personal (maintaining a threat-free, safe, and predictable personal environment).

We did not expect consistent, significant associations with global creativity for the remaining nine values that we measured. We next consider each specific domain of creativity and suggest reasons to expect variations in the motivating values for some specific domains. Table 4 summarizes the predictions for all 19 values.

**Table 4:** Predicted and observed associations between values and creativity.

	**Global creativity**		**Craft**		**Visual arts**		**Performance**		**Theater**		**Products for work**		**Machine graphics**	
**Value**	**Exp**.	**Obs**.	**Exp**.	**Obs**.	**Exp**.	**Obs**.	**Exp**.	**Obs**.	**Exp**.	**Obs**.	**Exp**.	**Obs**.	**Exp**.	**Obs**.
**Domains of Creativity**														
Self-Direction—thought	**+**	**+/+**	**+**	**+/+**	**+**	**+/+**	**+**	**+/+**	**+**	**+/+**	**+**	**+/+**	**+**	**+/+**
Self-Direction—action	**+**	**+/+**	**+**	**+/+**	**+**	**+/+**	**+**	**+/+**	**+**	**+/+**	**+**	**+/+**	**+**	**+/+**
Stimulation	**+**	**+/+**	**+**	**+**/o	+	**+/+**	**+**	**+**/o	**+**	**+/+**	**+**	**+/+**	**+**	**+/+**
Hedonism	**+**	**+/+**	**+**	o/o	+	**+/+**	**+**	**+**/o	**+**	**+/+**	**+**	**+/+**	**+**	**+/+**
Achievement	o	+/o	o	o/o	o	**+**/o	o	o/o	o	o/o	**+**	**+/+**	o	**+/+**
Power—dominance	o	o/o	**–**	**–/–**	o	o/o	o	o/o	o	o/o	o	o/o	o	o/o
Power—resources	o	o/o	**–**	**–/–**	o	o/o	o	**–/–**	o	o/o	o	o/o	o	o/o
Face	o	o/o	o	o/o	o	o/**–**	o	o/o	o	o/**–**	o	o/o	o	**–/–**
Security—personal	**–**	**–**/o	o	o/o	**–**	**–**/o	**–**	**–**/o	**–**	**–**/o	**–**	**–/–**	**–**	**–/–**
Security—societal	o	o/o	o	o/o	o	o/o	o	o/o	o	o/o	o	o/o	o	o/o
Tradition	**–**	**–/–**	**–**	**–/–**	**–**	**–/–**	**–**	o/**–**	**–**	**–/–**	**–**	**–/–**	**–**	**–/–**
Conformity—rules	**–**	**–/–**	**–**	**–/**o	**–**	**–/–**	o	o/o	**–**	**–/–**	**–**	**–/–**	**–**	**–/–**
Conformity—interpersonal	**–**	**–/–**	**–**	**–**/o	**–**	**–/–**	o	o/o	**–**	**–/–**	**–**	**–/–**	**–**	**–/–**
Humility	**–**	**–/–**	**–**	**–/–**	**–**	**–/–**	**–**	**–/**o	**–**	**–/–**	**–**	**–/–**	**–**	**–/–**
Universalism—nature	**+**	**+/+**	**+**	**+/+**	**+**	**+**/o	**+**	**+/+**	**+**	**+/+**	o	o/o	**+**	o/o
Universalism-concern	o	o/o	o	o/o	o	o/**–**	o	o/**–**	o	o/o	o	o/o	o	o/o
Universalism—tolerance	o	o/o	o	o/o	o	o/o	o	**+/–**	o	**+**/o	o	o/o	o	o/o
Benevolence—caring	o	**+/+**	**+**	**+/+**	o	o**/+**	**+**	o**/+**	o	o**/+**	o	**+/+**	o	o/+
Benevolence—dependability	o	o/o	**+**	**+/+**	o	o/o	**+**	o**/+**	o	o/o	o	**+/+**	o	o/+

*Exp., expected association. Obs., observed association. **+**, positive association; –, negative association; o, no association*.

*Observed results precede the slash mark for Central Russia and follow it for the North Caucasus*.

### Craft

The crafts measured here included decorative activities with simple materials (e.g., beads, flowers, clothe, and glass) and designing or making costumes and clothing by sewing, knitting, or embroidering. Making clothing, costumes, and decorative articles is often performed by parents for or with their children. These activities often occur in families or with friends. They can be enjoyed even without high levels of skill and entail little risk of failure or public criticism. The supportive, in-group atmosphere and low likelihood of failure suggest that personal security values will not inhibit engaging in crafts. Moreover, the in-group, sociable ambience of most craft activities suggests that valuing Benevolence-Caring and Benevolence-Dependability may motivate engaging in them for one's in-group (Schwartz, 2010). Dollinger et al. (2007), who used a craft-heavy index of creativity, reported a negative association with Power values in one study, perhaps because crafts activities offer little opportunity to exercise control over others or over wealth. This suggests a negative association with Power-Dominance and Power-Resources for craft creativity.

### Visual Arts

The visual art activities measured here included painting an original picture, sculpture, and sketching. These activities typically require some talent and at least moderate levels of skill. A meta-analysis of the personality characteristics of artists compared with non-artists by Feist (1998) found that artists (mostly painters and sculptors) tend to be more aesthetic, curious, imaginative, impulsive, and open to experience. They also tend to be less cautious, concerned with making a good impression, conscientious, conventional, conformist, reliable, socialized, and warm than non-artists. This summary, based on findings with several different personality scales, suggests that the same values expected to predict global creativity should also predict creativity in the visual arts.

### Performance

The performance activities measured here included singing, dancing, and playing musical instruments in public, often as part of an ensemble. Like the visual arts, these activities require some talent and at least moderate levels of skill. The inhibiting values relevant to global creativity are likely to predict performance too, with the possible exception of Conformity-Rules and Conformity-Interpersonal. Most of the performances measured require conforming to the requirements of an ensemble (e.g., orchestra, band, chorus, dance troupe), so the motivation to conform need not oppose engaging in these activities. The group nature of much performance also suggests that Benevolence-Caring and Benevolence-Dependability values may motivate it.

### Theater

The theater activities measured here included directing or acting in a theatrical performance and putting on a puppet show. Despite the group nature of much theatrical activity, we do not expect Benevolence values to promote it because each actor or director has a unique role in which they are likely to pursue their own performance goals. We expect that the same values that predict global creativity should also predict creativity in theater.

### Products for Work

The work products measured here included developing new hardware, software, procedures, designs, and presentation materials in one's work setting. All of the values expected to predict global creativity appear relevant for predicting creativity in this domain with one exception: Universalism-Nature presumably promotes creativity because it encourages the aesthetic aspect that is not a focus of most of the activities in this domain. We also expected Achievement values to promote creativity in this domain because producing new products in one's work setting is a way to advance up the ladder of success.

### Machine Graphics

The machine graphic items included making movies and using computers to draw pictures, cartoons, or collages and make websites. The same values expected to predict global creativity appear relevant for predicting creativity in this domain.

### Method

#### Values

To measure individuals' basic values, we administered the revised Portrait Value Questionnaire (PVQ-R: Schwartz et al., 2012; Schwartz, 2017). It measures the 19 distinct values in the refined theory of basic values. The PVQ-R includes 57 items (3 items for each value) that describe different people in terms of valued goals that are important to them. Respondents indicate how similar each person is to themselves on a 6-point scale ranging from 1 (not like me at all) to 6 (very much like me). We infer respondents' own values from the values of the people they say are similar to themselves. The PVQ-R was translated into Russian using double reverse translation with native English and Russian speakers. Schwartz and Butenko (2014) reported findings that supported the validity and reliability of the Russian version.

We tested the cross-region invariance of all 19 value scales. For each scale we conducted a multigroup confirmatory factor analysis. As each scale has three items, the configural invariance model cannot be tested because it has zero degrees of freedom and a perfect fit by definition. Table 5 presents the fit statistics of the metric and scalar invariance model. Metric invariance is supported in all cases, except for Humility, where the estimation process did not converge. Scalar invariance is supported for most scales, except for Self-Direction—Thought, Stimulation, Achievement, and the Universalism values. We concluded that scores can be compared for most scales across regions, although care is needed when interpreting differences on scales that showed only metric invariance.

**Table 5:** Invariance of the 19 values scales.

**Value**	**Metric invariance**			**Scalar invariance**				
	**χ^2^/*df*^a^**	**CFI**	**RMSEA**	**χ^2^/*df*^b^**	**Δχ^2^/Δ*df*^a^**	**CFI**	**ΔCFI**	**RMSEA**
Self-direction—thought	0.31	1.000	0.000	2.64^*^	4.97^**^	0.803	0.197	0.041
Self-direction—action	6.12^**^	0.964	0.073	4.54^**^	2.96	0.950	0.014	0.060
Stimulation	2.89	0.975	0.044	4.23^**^	5.56^**^	0.915	0.060	0.058
Hedonism	0.49	1.000	0.000	4.26^**^	8.03^***^	0.981	0.019	0.058
Achievement	2.92	0.986	0.045	4.67^***^	8.43^***^	0.933	0.054	0.070
Power—dominance	2.17	0.998	0.035	1.11	0.06	1.000	−0.002	0.011
Power—resources	4.82^**^	0.988	0.063	2.47^*^	0.13	0.990	−0.002	0.039
Face	2.06	0.994	0.033	1.16	0.26	0.998	−0.004	0.013
Security—personal	0.50	1.000	0.000	1.84	3.20^*^	0.982	0.018	0.030
Security—societal	5.68^**^	0.980	0.070	3.40^**^	1.13	0.979	0.001	0.050
Tradition	0.27	1.000	0.000	0.69	1.11	1.000	0.000	0.000
Conformity—rules	2.78	0.995	0.043	1.45	0.11	0.998	0.003	0.021
Conformity—interpersonal	0.42	1.000	0.000	0.74	1.05	1.000	0.000	0.000
Humility	No convergence			1.53	NA	0.971	NA	0.023
Universalism—nature	2.08	0.995	0.033	5.66^***^	9.24^***^	0.959	0.036	0.069
Universalism—concern	12.22^***^	0.929	0.108	11.08^***^	9.94^***^	0.873	0.049	0.102
Universalism—tolerance	1.11	0.999	0.010	1.73	2.35	0.980	0.019	0.027
Benevolence—caring	1.25	0.999	0.016	1.49	1.74	0.997	0.002	0.022
Benevolence—dependability	4.46^*^	0.983	0.060	3.95^**^	1.44	0.980	0.003	0.045

*Configural invariance could not be tested because each scale had three items, which yields a perfect fit in a test of configural invariance*.

a *df = 2*.

b *df = 4*.

* *p < 0.05*.

** *p < 0.01*.

*** *p < 0.001*.

#### Creativity

For each of the six creativity domains and for global creativity, we used the factor score derived from the loadings on the respective latent creativity variable in Study 1. This scoring approach adopts the hybrid model supported in Study 1.

#### Socio-demographic Variables

We computed age from year of birth. Interviewers coded gender 1 (male) or 2 (female). Respondents reported their highest level of completed education on a scale from 1 (basic secondary education) to 11 (academic degree stage II PhD). Respondents reported whether they considered themselves as belonging to a particular religion or denomination. Almost all reported either Russian Orthodox, Muslim, or no religion. We created pacifier variables for Russian Orthodox and Muslim, with unaffiliated as the reference category. Religiosity was measured on an 11-point self-report scale from 0 (not at all religious) to 10 (very religious).

### Results

#### Descriptive Findings

Table 6 presents the means and standard deviations of the domains of creative behavior and of values by region. To compare regions, we ran a MANOVA on all variables. The last column of Table 6 presents effect sizes (Cohen's *d)*. For all six domains, the level of creativity was greater in Central Russia, with moderate effect sizes. For values, the regions differed significantly on 16 of the 19 values (for values that did not show scalar invariance, these results should be interpreted with caution). Openness to Change values, especially Self-Direction and Hedonism, were higher in Central Russia. Conservation and Self-Transcendence values, especially Conformity-Rules, Tradition, Humility, and Face, were higher in the North Caucasus.

**Table 6:** Means and standard deviations of creativity and values by region and effect sizes (d) for regional differences.

**Scale**	**Central Russia**	**North Caucasus**	**Cohen's *d***
**CREATIVITY**			
Global	1.26 (0.36)	1.12 (0.27)	0.44^***^
Crafts	1.61 (0.82)	1.28 (0.55)	0.47^***^
Visual arts	1.26 (0.57)	1.12 (0.37)	0.29^***^
Performance	1.32 (0.60)	1.19 (0.48)	0.24^***^
Theater	1.18 (0.43)	1.09 (0.35)	0.23^***^
Products for work	1.31 (0.54)	1.12 (0.32)	0.43^***^
Machine graphics	1.29 (0.51)	1.15 (0.38)	0.31^***^
**HIGHER-ORDER VALUES**			
Openness	4.04 (0.44)	3.88 (0.46)	0.36^***^
Self-enhancement	3.72 (0.54)	3.70 (0.57)	0.04
Conservation	4.02 (0.39)	4.12 (0.38)	−0.26^***^
Self-transcendence	4.15 (0.34)	4.18 (0.34)	−0.09^*^
**BASIC VALUES**			
Self-direction—thought	4.32 (0.53)	4.16 (0.56)	0.29^***^
Self-direction—action	4.29 (0.63)	4.19 (0.63)	0.16^**^
Stimulation	3.65 (0.83)	3.48 (0.81)	0.21^***^
Hedonism	3.92 (0.84)	3.68 (0.92)	0.27^***^
Achievement	3.80 (0.79)	3.80 (0.77)	0.00
Power—dominance	3.10 (1.13)	2.99 (1.21)	0.09^*^
Power—resources	3.61 (0.88)	3.49 (1.00)	0.13^**^
Face	4.38 (0.62)	4.53 (0.56)	−0.25^***^
Security—personal	4.43 (0.62)	4.35 (0.56)	0.14^**^
Security—societal	4.48 (0.71)	4.32 (0.67)	0.23^***^
Tradition	3.98 (0.72)	4.20 (0.69)	−0.31^***^
Conformity—rules	3.72 (0.86)	3.99 (0.77)	−0.33^***^
Conformity—interpersonal	3.80 (0.74)	3.94 (0.78)	−0.18^***^
Humility	3.69 (0.77)	3.89 (0.69)	−0.27^***^
Universalism—nature	3.75 (0.79)	3.84 (0.76)	−0.12^**^
Universalism—concern	4.14 (0.66)	4.18 (0.65)	−0.06
Universalism—tolerance	3.78 (0.75)	3.91 (0.68)	−0.18^***^
Benevolence—caring	4.57 (0.59)	4.48 (0.53)	0.16^***^
Benevolence—dependability	4.53 (0.58)	4.49 (0.51)	0.07

*Note. Values are within-person centered*.

* *p < 0.05*.

** *p < 0.01*.

*** *p < 0.001*.

#### Associations of Creativity With Background Characteristics

A set of regressions examined effects of gender, age, education, religiosity, and religion on global creativity and on each of the six domains of creativity. Because region affected all domains in the same direction and none of its interactions with the other background variables explained meaningful variance, we did not include it in the regressions. Table 7 reveals that creativity was greater among women than men in the crafts and performance domains but greater among men than women in the products for work and machine graphics domains. Younger respondents reported more creativity than older in global creativity and in the visual arts, theater, and machine graphics domains. Higher education was associated with greater creativity in all domains except performance. Religiosity predicted creativity only in the crafts domain. Being Muslim was the strongest negative predictor of creativity, overall and in every domain, but the other two religious groups did not differ in creativity.

**Table 7:** Regression analysis (Standardized Coefficients) with background characteristics as predictors and creativity domains as dependent variables.

	**Predictor**						
**Creativity domain**	**Gender^a^**	**Age**	**Education**	**Religiosity**	**Orthodox^b^**	**Muslim^b^**	***R*^**2**^**
Global	0.02	−0.08^***^	0.10^***^	0.03	0.01	−0.19^***^	0.05^***^
Crafts	0.17^***^	0.03	0.10^***^	0.05^*^	0.01	−0.24^***^	0.10^***^
Visual arts	0.01	−0.08^***^	0.11^***^	0.04	−0.03	−0.20^***^	0.05^***^
Performance	0.05^*^	−0.03	0.02	−0.01	−0.02	−0.22^***^	0.05^***^
Theater	0.03	−0.05^*^	0.06^**^	0.02	−0.02	−0.20^***^	0.04^***^
Products for work	−0.06^**^	−0.03	0.18^***^	0.05	−0.01	−0.21^***^	0.08^***^
Machine graphics	−0.09^***^	−0.11^***^	0.12^***^	0.03	−0.02	−0.20^***^	0.08^***^

a *1, male, 2, female*.

b *Unaffiliated as reference category*.

* *p < 0.05*.

** *p < 0.01*.

*** *p < 0.001*.

#### Associations of Creativity and Values

To test our hypotheses regarding relations of creativity to values, we correlated factor scores for the six domains and global creativity with sum scores for the four higher order values and 19 basic values. We used correlation analysis for each value separately because substantial inter-correlations among them are inherent in their location on a circular motivational continuum. Separate correlations avoid the impact of high multicollinearity among the values and reveal the distinct relations of each value.

As expected, the higher order Openness to Change value correlated positively with global creativity in both Central Russia (*r* = 0.26, *p* < 0.001) and the North Caucasus (*r* = 0.21, *p* < 0.001). Also as expected, the higher order Conservation value correlated negatively with global creativity in both regions (*r* = −0.24, *p* < 0.001, *r* = −0.21, *p* < 0.001, respectively). The expected weak positive association for the higher order Self-Transcendence value was present in Central Russia (*r* = 0.08, *p* < 0.05) but not in the North Caucasus (*r* = 0.04, *p* > 0.05) As expected, the higher order Self-Enhancement value was unrelated to global creativity in either region (*r* = 0.00, *r* = 0.00). Table A in the Online Supplement lists the correlations of the higher order values with each of the creativity domains.

We next examine correlations of each of the 19 basic values with global creativity and with creativity in each domain. See Table 4, above, for a summary of the expected and observed directions of the correlations.

##### Global Creativity

In both regions, global creativity correlated positively with Self-Direction-Thought, Self-Direction-Action, Stimulation, Hedonism, and Universalism-Nature, and negatively with Tradition, Conformity-Rules, Conformity-Interpersonal, and Humility, as expected (see Table 8). The expected negative correlation with Security-Personal emerged only in Central Russia. In addition, there were unexpected positive correlations with Achievement in Central Russia and with Benevolence-Caring in both regions.

**Table 8:** Correlations of 19 basic values with global creativity.

**Value**	**Central Russia**	**North Caucasus**
Self-direction—thought	0.18^***^	0.15^***^
Self-direction—action	0.12^***^	0.12^***^
Stimulation	0.16^***^	0.09^**^
Hedonism	0.11^**^	0.08^*^
Achievement	0.08^**^	0.03
Power—dominance	−0.03	0.01
Power—resources	−0.01	−0.02
Face	−0.04	−0.04
Security—personal	−0.08^**^	−0.03
Security—societal	0.00	0.01
Tradition	−0.10^**^	−0.12^***^
Conformity—rules	−0.14^***^	−0.13^***^
Conformity—interpersonal	−0.14^***^	−0.10^**^
Humility	−0.17^***^	−0.12^***^
Universalism—nature	0.08^*^	0.09^**^
Universalism—concern	−0.03	−0.04
Universalism—tolerance	0.04	−0.04
Benevolence—caring	0.07^*^	0.11^***^
Benevolence—dependability	0.06	0.03

* *p < 0.05*.

** *p < 0.01*.

*** *p < 0.001*.

##### Crafts

We expected the value predictors of global creativity, with the exception of Security-Personal, to predict craft creativity too. In addition, we expected the two Benevolence values to promote craft creativity and the two Power values to inhibit it. The correlations in Table 9 for Central Russia support all the predictions except for a lack of association between Hedonism values and creativity. In the North Caucasus, only nine of the 13 predictions were supported: Neither Hedonism nor Stimulation values promoted craft creativity and neither Conformity-Rules nor Conformity-Interpersonal values inhibited it.

**Table 9:** Correlations of 19 basic values with craft and visual art creativity.

	**Craft**		**Visual arts**	
**Value**	**Central Russia**	**North Caucasus**	**Central Russia**	**North Caucasus**
Self-direction—thought	0.17^***^	0.15^***^	0.19^***^	0.14^***^
Self-direction—action	0.09^**^	0.09^**^	0.17^***^	0.12^***^
Stimulation	0.10^**^	0.03	0.17^***^	0.10^**^
Hedonism	0.02	0.00	0.09^**^	0.07^*^
Achievement	0.03	−0.06	0.10^**^	0.05
Power—dominance	−0.12^***^	−0.07^*^	−0.02	0.04
Power—resources	−0.08^**^	−0.08^*^	−0.03	0.01
Face	0.00	0.00	*−0.02*	−0.09^**^
Security—personal	0.01	0.03	−0.07^*^	−0.03
Security—societal	0.01	0.10^**^	−0.04	−0.02
Tradition	−0.07^*^	−0.08^**^	−0.15^***^	−0.11^***^
Conformity—rules	−0.08^**^	−0.04	−0.10^**^	−0.13^***^
Conformity—interpersonal	−0.07^*^	−0.03	−0.16^***^	−0.09^**^
Humility	−0.16^***^	–0.07^*^	−0.17^***^	–0.09^**^
Universalism—nature	0.13^***^	0.11^***^	0.06^*^	0.01
Universalism—concern	−0.02	−0.04	−0.03	−0.07^*^
Universalism—tolerance	0.05	−0.01	0.01	−0.01
Benevolence—caring	0.11^***^	0.12^***^	0.06	0.10^**^
Benevolence—dependability	0.09^**^	0.09^**^	0.04	0.04

*Correlations printed in italics differ significantly across the regions in a bootstrapped procedure, using 1,000 samples (p < 0.05)*.

* *p < 0.05*.

** *p < 0.01*.

*** *p < 0.001*.

##### Visual arts

We had the same 10 predictions for visual arts as for global creativity. In Central Russia, these predictions were confirmed (see Table 9). In the North Caucasus, eight predictions were confirmed: The associations suggest that Security-Personal values do not inhibit creativity and Universalism-Nature values do not promote it. In addition, Face values related negatively and Benevolence-Caring values related positively to visual arts creativity.

##### Performance

We expected the value predictors of global creativity, with the exception of the two Conformity values, to predict performance creativity. In addition, we expected the two Benevolence values to promote performance creativity. The correlations in Table 10 supported seven of the 10 predictions in Central Russia: The correlations suggest that tradition values do not inhibit performance creativity and both Benevolence values do not promote it. In the North Caucasus, only six of the 10 predictions were supported: Neither Hedonism nor Stimulation values related positively to creativity and neither Security-Personal nor Humility values related to it negatively. In both regions, unexpectedly, Power Resources values related negatively to performance creativity

**Table 10:** Correlations of 19 basic values with performance and theater creativity.

	**Performance**		**Theater**	
**Value**	**Central Russia**	**North Caucasus**	**Central Russia**	**North Caucasus**
Self-direction—thought	0.13^***^	0.14^***^	0.14^***^	0.08^**^
Self-direction—action	0.07^*^	0.09^**^	0.11^**^	0.08^*^
Stimulation	0.11^***^	*0.01*	0.18^***^	0.07^*^
Hedonism	0.09^**^	0.04	0.12^***^	0.07^*^
Achievement	0.00	−0.04	0.06	0.00
Power—dominance	−0.05	−0.02	−0.02	−0.01
Power—resources	−0.10^**^	−0.09^**^	−0.06	−0.02
Face	−0.05	−0.01	−0.05	−0.06^*^
Security—personal	−0.09^**^	*0.04*	−0.11^***^	*-0.02*
Security—societal	−0.03	0.01	−0.06	0.01
Tradition	−0.05	−0.07^*^	−0.08^**^	−0.07^*^
Conformity—rules	−0.04	−0.04	−0.10^**^	−0.08^*^
Conformity—interpersonal	−0.05	−0.04	−0.11^***^	−0.07^*^
Humility	−0.10^**^	−0.05	−0.16^***^	−0.07^*^
Universalism—nature	0.09^**^	0.09^**^	0.06^*^	0.07^*^
Universalism—concern	*0.05*	–0.09^**^	−0.01	−0.02
Universalism—tolerance	0.07^*^	−0.07^*^	0.09^**^	−0.02
Benevolence—caring	*0.03*	0.13^***^	0.03	0.08^*^
Benevolence—dependability	*0.01*	0.12^***^	0.03	0.03

*Correlations printed in italics differ significantly across the regions in a bootstrapped procedure, using 1,000 samples (p < 0.05)*.

* *p < 0.05*.

** *p < 0.01*.

*** *p < 0.001*.

##### Theater

We had the same 10 predictions for theater as for global creativity. In Central Russia, these predictions were confirmed. In addition, Universalism-Tolerance values related positively to theater creativity (see Table 10). In the North Caucasus, nine predictions were confirmed: Security-Personal values were unrelated to creativity. In addition, the correlations suggest that Benevolence –Caring values promote theater creativity and Face values inhibit it.

##### Products for work

We expected the value predictors of global creativity, with the exception of Universalism-Nature values, to be associated with creativity in products for work. We also expected Achievement values to be related to this type of creativity. The correlations in Table 11 supported all 10 of these predictions in both Central Russia and the North Caucasus. In addition, both types of Benevolence values related positively to creativity in products for work.

**Table 11:** Correlations of 19 basic values with products for work and machine graphics creativity.

	**Products for work**		**Machine graphics**	
**Value**	**Central Russia**	**North Caucasus**	**Central Russia**	**North Caucasus**
Self-direction—thought	0.22^***^	0.17^***^	0.20^***^	0.14^***^
Self-direction—action	0.19^***^	0.17^***^	0.19^***^	0.13^***^
Stimulation	0.18^***^	0.12^***^	0.21^***^	0.12^***^
Hedonism	0.08^**^	0.09^**^	0.15^***^	0.11^***^
Achievement	0.12^***^	0.07^*^	0.13^***^	0.08^**^
Power—dominance	−0.02	0.05	0.02	0.06
Power—resources	0.02	0.01	0.02	0.02
Face	−0.05	−0.03	−0.06^*^	−0.06^*^
Security—personal	−0.09^**^	−0.08^*^	–0.14^***^	–0.07^*^
Security—societal	−0.03	0.02	−0.05	−0.03
Tradition	−0.10^**^	−0.14^***^	−0.13^***^	−0.13^***^
Conformity—rules	−0.14^***^	−0.16^***^	−0.16^***^	−0.17^***^
Conformity—interpersonal	−0.18^***^	−0.16^***^	−0.20^***^	−0.14^***^
Humility	−0.19^***^	−0.15^***^	−0.19^***^	−0.13^***^
Universalism—nature	0.02	−0.04	0.00	−0.01
Universalism—concern	−0.09	0.00	−0.06	−0.04
Universalism—tolerance	0.02	−0.06	0.03	−0.05
Benevolence—caring	0.08^*^	0.09^**^	0.04	0.09^**^
Benevolence—dependability	0.09^**^	0.08^*^	0.06	0.07^*^

*Correlations printed in italics differ significantly across the regions in a bootstrapped procedure, using 1,000 samples (p < 0.05)*.

* *p < 0.05*.

** *p < 0.01*.

*** *p < 0.001*.

##### Machine graphics

We expected the value predictors of global creativity to predict creativity in machine graphics. The correlations in Table 11 supported all 10 of these predictions in both Central Russia and the North Caucasus. In addition, in both regions, the correlations suggest that Achievement values and both types of Benevolence values promote creativity in machine graphics.

### Discussion

#### Background Variables and Creativity

Region was the strongest predictor of creativity scores. For all six domains, creativity was greater in Central Russia than in the North Caucasus. This finding was hardly surprising. As noted in the introduction, the North Caucasus region has a more traditional culture. It is characterized by higher religiosity, stronger ethnic identification, larger and more closely knit families than the Central region, and it is more agricultural and less urbanized and industrialized (Orttung, 2000; O'Loughlin et al., 2009; Kilinkarova, 2013). Cultural expectations and social structural constraints that support traditional ways of doing things discourage the innovation and unconventional behavior needed for creativity. In contrast, cultural expectations and social structural opportunities grounded in more rapid change, weaker family ties, greater personal freedom, and tolerance for unconventional and unique behavior in the more urbanized and industrialized Central region may facilitate the climate that fosters creativity (Florida, 2005; Adam and Westlund, 2013).

In the terms of a recent paper (Chiu et al., 2018), the culture of the Central Russia region is likely to socialize for greater “Self-Directedness” and that of the North Caucasus for greater “Other-Directedness.” Socialization differences on this dimension explain substantial variance in the creativity of one's job engagement across 50 nations. Our finding of creativity differences across two cultures in a variety of domains fits this general cross-national pattern.

The strongest background predictor of creativity was religion: Muslim respondents were significantly less creative than Russian Orthodox or unaffiliated respondents on both global creativity and creativity in all domains. This might be attributed to more restrictive views of various art forms in Islam, but this may not be the only or even most likely factor. Restrictions in Islam concern depiction of living forms (Nasr, 1987; Dollinger, 2007). They cannot explain low creativity in most of the domains studied here (e.g., crafts or products for work). Rather, this finding probably reflects the fact that 98% of the Muslims in this study lived in the more traditional, North Caucasus region. Thus, this effect of religion is largely confounded with the effect of region.

Greater education promoted all types of creativity except performance. This may reflect the benefits of the knowledge and skills obtained through education for creativity. It is also possible that more creative people are more likely to pursue higher education both because their creativity makes them more successful and because they are more confident that they will be able to use what they learn effectively. It is unclear why education had no effect in the performance domain.

Each of the specific domains of creativity exhibited a somewhat distinctive pattern of influence by the other background variables. Gender was the strongest predictor of crafts, followed by education and religiosity. Not surprisingly, women engaged more in activities such as embroidering and making costumes and decorations. Crafts may be linked to religiosity because religious ceremonies often call for such activity. In fitting with traditional gender roles (Deaux and Lewis, 1984; Kerig et al., 1993), female respondents engaged in crafts more than male respondents did. Religion may be an important moderator of engagement in crafts. Russian Orthodox respondents engaged more in crafts than the religiously unaffiliated. This could be a consequence of the link between religion and focus on tradition. In addition, the stronger engagement of Russian Orthodox could reflect the historic association of Russian Orthodoxy with creativity in the decorative arts, including embroidery of icons, mosaics and stained glass in churches, and the manufacture of objects of worship from simple materials (Nikolaeva, 1976).

Education was the strongest predictor of visual arts creativity, followed by age. Younger people were a little more likely to paint or sculpt. Only gender predicted performance significantly but weakly. Women were a little more likely to sing, dance, or play musical instruments. More educated and younger people were slightly more likely to engage in theatrical activities. Education had its strongest effect on products for work (new machines, hardware/software, architectural plans, work procedures), the type of creativity that required the greatest formal training. Men were also slightly more likely to engage in this type of creativity. Education, age (younger), and gender (male) related positively to machine graphics (working with computers to create various graphics or making movies). Such activity, with its heavy use of computers, often benefits from formal training and attracts younger people who grew up feeling comfortable with technology (Czaja et al., 2006), more frequently males.

#### Values and Creativity

##### Global creativity

Based on past findings and on analyses of the consequences of creative activity for the motivational goals of the 19 basic values in the Schwartz et al. (2012) theory, we derived expectations for the associations between values and creativity. We hypothesized that the four Openness to Change values (Self-Direction-Thought, Self-Direction-Action, Stimulation, and Hedonism) would promote global creativity because these values motivate self-expansive, novelty seeking activity. We further postulated that five of the Conservation values (Security-Personal, Tradition, Conformity-Rules, Conformity-Interpersonal, and Humility) would inhibit global creativity because these values motivate self-protective, self-restrictive activity that maintains a stable and predictable environment. Confirmation of all these hypotheses strengthens our reasoning about the motivations underlying creativity^1^. Also confirmed was our reasoning that Universalism-Nature values promote global creativity because these values motivate appreciation of aesthetics, which finds expression in most domains of creativity studied here.

Unexpectedly, Benevolence-Caring values also promoted global creativity and, in one or both regions, creativity in all of the specific domains. Benevolence-Caring values, involving devotion to the welfare of in-group members, may encourage participation in activities that involve cooperation with family and friends, activities of everyday creativity (e.g., crafts, performance in ensembles). Benevolence-Caring values may also encourage contributing creatively to the goals of organizations and of peers at work (e.g., products for work, machine graphics). Work on organizational citizenship behavior has identified benevolence values as promoting behavior that contributes to work teams (Arthaud-Day et al., 2012).

We next discuss relations of values to the specific domains of creativity. We do not discuss findings for the four Openness to Change values, the five Conservation values, and Universalism-Nature values unless the predictions or the results differed from those for global creativity.

##### Crafts

As distinct from global creativity, we expected both types of Benevolence values to promote craft creativity because crafts are often performed by parents with or for their children or together with friends. Moreover, we expected no inhibiting effect of Security-Personal values because the threat of failure is small in such settings. Findings in both regions supported this reasoning. Only in the North Caucasus, contrary to our hypotheses, Stimulation and Hedonism values failed to promote craft activity and both types of Conformity values failed to inhibit it. This suggests that the settings for most craft activities in the North Caucasus are even more comfortable, low keyed, and less judgmental of others' creations than in Central Russia. Crafts apparently provide minimal sensual pleasure compared with other types of creativity, because Hedonism promoted all the types of creativity except crafts in Central Russia.

##### Visual arts

In Central Russia, the values expected to predict global creativity all predicted creativity in the visual arts. All but Security-Personal and Universalism-Nature values did so in the North Caucasus. In addition, Achievement values promoted creativity in Central Russia. Benevolence-Caring values promoted creativity in the North Caucasus whereas Face values inhibited it. These findings suggest somewhat different perceptions of activities like sculpture and painting in the two regions. They may be seen as relatively informal hobbies pursued among friends in the traditional culture of the North Caucasus. In contrast, they may be seen as more ambitious, formal aesthetic pursuits that express individual talent in the more modernized Central Russian environment.

##### Performance

In contrast to global creativity, we expected no inhibition of performance creativity by the two Conformity values. The results confirmed our expectations. We based the Conformity hypotheses on the assumption that most performance activities in our samples occur in non-professional ensembles. Supporting this assumption, no one reported an occupation as a performer. The Conformity values results support our reasoning that performing non-professionally in ensembles implies willingness to conform to group norms and is unlikely to be perceived as unconventional.

We also expected both Benevolence values to promote performance creativity. They did so in the North Caucasus. This supports our reasoning that valuing Benevolence makes one more comfortable cooperating closely with an in-group, as required when participating in ensembles. The close, in-group atmosphere may also explain why Personal Security values did not inhibit creativity in the North Caucasus. In Central Russia, the two Benevolence values failed to promote performance, contrary to expectations, and Personal Security inhibited it. This finding is compatible with the view that performance, like visual arts, is seen in the more modernized Central Russian environment as an expression of individual talent. Hence, the possibility of failure constitutes a personal threat.

Power-Resources values inhibited performance creativity in both regions. This may reflect an understanding that wealth is unlikely to accrue from engaging in performance as a non-professional musician or dancer. The remaining values that predicted global creativity also predicted performance, with the exception of Stimulation and Hedonism values in the North Caucasus. Moreover, both Universalism-Concern and Universalism-Tolerance inhibited performance creativity in the North Caucasus and Humility did not. The presence of five unexpected findings for performance creativity in the North Caucasus is puzzling. Rather than offer multiple speculations, we look to future research for explanations.

##### Theater

We expected all the value predictors of global creativity to predict creativity in the theater domain. Findings supported this expectation in both regions with a single exception in the North Caucasus. There, Personal Security values failed to inhibit participation but Face values did inhibit it. As with performance creativity, the close, in-group atmosphere in the North Caucasus may also explain why Personal Security values did not inhibit creativity in the theater domain. It may also explain why Benevolence-Caring values did promote creativity. Face related negatively to creativity in both regions but reached significance only in the North Caucasus where Face is the most important of the 19 values (see Table 6). Concern with losing Face when appearing on stage may therefore have inhibited this type of creativity. We have no explanation for why Universalism-Tolerance promoted theater creativity in Central Russia.

##### Products for work

With the exception of Universalism-Nature, we expected all the value predictors of global creativity to predict creativity in products for work. We excepted Universalism-Nature because the work products listed here minimally concern aesthetics. We also expected Achievement values to predict this type of creativity because producing new products in one's work setting is a way to advance up the ladder of success. Supporting our reasoning, these expectations were all confirmed. In addition, both Benevolence values promoted creativity in both regions. Benevolence-Caring, by encouraging cooperative behavior, likely influences creativity indirectly through motivating people to contribute to the goals of organizations and of work peers. Benevolence-Dependability probably promotes creativity at work because it motivates being a reliable and trustworthy member of the in-group—a good organizational citizen (cf. Arthaud-Day et al., 2012).

##### Machine graphics

As expected, all of the values that predicted global creativity predicted creativity in the machine graphics domain in both regions. Moreover, Achievement values promoted creativity in both regions. This suggests that, similar to products for work, creating machine graphics is a way to advance up the ladder of success. Two of the three machine graphics items specify that the graphic product was intended “to show other people.” In the North Caucasus, in addition, both Benevolence values promoted this type of creativity. This suggests that this type of creativity is often carried out as a contribution to the in-group in the North Caucasus.

Of the total of 63 hypothesized associations of values with creativity in the six domains, 58 were confirmed in the Central Russia sample and 51 in the North Caucasus sample. In addition, there were six unexpected significant associations in Central Russia and 14 in the North Caucasus. Comparing the correlations in the two regions in all instances in which there was a significant association in at least one region, 62% were higher in Central Russia, 9% were the same, and only 23% were higher in the North Caucasus. This pattern of stronger association between values and creative behavior in Central Russia may be due to the smaller variance in the behaviors in the North Caucasus (Table 6).

More interestingly, the stronger associations in Central Russia than in the North Caucasus may reflect effects of culture on the value-creativity relationship. Church et al. (2006) have found that behavior is more “traited” (more under the individual's control) in more modern cultural contexts and is more situationally controlled in more traditional contexts. Along the same lines, the current data suggest that the link between motivational factors (values) and creative behavior is stronger in a more modern cultural context than in a more traditional cultural context. Similarly, the data suggest that personal values guide behavior more in contexts that emphasize self-directedness (cf. Chiu et al., 2018). There, people are freer to act on their own values because they have the economic resources to do so (for a similar argument regarding value-attitude links, see Boer and Fischer, 2013).

## Conclusion

Study 1 addressed the question of whether creativity is domain-general or domain-specific. We adopted a hybrid point of view, positing that there are both domain specific and general characteristics of creativity (Plucker and Beghetto, 2004). We distinguished several potential domains of creative activity that could be measured with a questionnaire modified from Dollinger (2003). We posited that it would be possible to distinguish these domains in the responses of two representative samples from Russian regions, using multi-group confirmatory factor analysis. We further posited that these domains would all load on a higher order global creativity factor. The analyses supported these expectations. The factor analysis distinguished six domains of creative behavior, all of which loaded on the same higher order, general, factor. We labeled the domains: crafts, visual arts, performance, theater, products for work, and machine graphics. This is one of the first empirical demonstrations of the hybrid view.

Study 2 examined individual characteristics that predict the frequency of creative activity. First, we considered possible effects of region, gender, age, education, religiosity, and religious affiliation on creativity in each domain of creativity. Region and religious affiliation had similar effects on all types of creativity. The other background variables had relatively weak effects on creativity. Nonetheless, they provided distinct profiles of predictors for creativity in each domain. This reinforced the hybrid view of creativity which recognizes distinctive types of creativity that are all related to a latent global creativity factor.

The main focus of Study 2 was to examine the motivations that propel and inhibit the different types of creativity. We used individuals' personal value priorities to measure motivations. We grounded a set of expectations regarding the values that would motivate each type of creative behavior in two sources. First were findings in past studies of values and creativity. Second were analyses of the consequences of engaging in creative behavior for the attainment of the motivational goals of each value. We asked whether creativity would serve these goals or would undermine their attainment. On these bases, we postulated that Openness to Change values (Self-Direction-Thought, Self-Direction-Action, Stimulation, and Hedonism) together with Universalism-Nature values would promote global creativity and that Conservation values^2^ (Security-Personal, Tradition, Conformity-Rules, Conformity Interpersonal, and Humility) would inhibit global creativity. Correlation analyses supported all of these expectations and revealed that Benevolence-Caring values also promoted global creativity.

We then considered whether creative activity in each of the six specific domains would serve the motivational goals of the values or would undermine them. This suggested that some values that predict global creativity were not relevant in particular domains while others were relevant. We specified these variations from the predictors of global creativity for each domain. Findings supported these expectations in most, but not all cases. We also found that creativity was higher in the Central Russian region than in the North Caucasus for all domains. The variation across domains in the predictors of creativity attested to the benefit of discriminating among domains.

This study went beyond past research in several ways. First, we found that Benevolence-Caring values, overlooked in past studies, consistently promoted creativity. We attributed this to the readiness of those who value Benevolence-Caring to cooperate in everyday creative activities with family and friends and to contribute creatively to their organizations. Benevolence-Dependability also promoted creativity in several domains. We attributed this to the motivation of those who value Benevolence-Dependability to contributing to their in-groups as reliable and trustworthy group members. Thus, the Benevolence values do not motivate creativity directly; they do so by motivating cooperative, helpful, and contributing behavior that promotes creativity.

Second, we found that only the two Self-Direction values promoted creativity and only Tradition, Conformity-Rules, and Humility values inhibited creativity across all or almost all domains in both regions. In contrast, the effects of Stimulation, Hedonism, Security-Personal, and Universalism-Nature and both Power values were domain dependent.

Third, by applying the refined theory of basic values, we identified two previously unrecognized values that consistently inhibited creativity. First, Humility, whose motivational goal is to recognize one's insignificance in the larger scheme of things and to avoid drawing attention to oneself, inhibited creativity in every domain except performance in the North Caucasus. Second, Face, whose motivational goal is to maintain one's public image and avoid humiliation, inhibited creativity in three domains.

Finally, the refined theory also enabled us to specify more precisely the components of Universalism and Security values that relate to creativity. Only Universalism-Nature, the aesthetic facet of universalism, consistently promoted creativity and only Personal rather than Societal Security inhibited creativity.

## Implications and Limitations

Our study has various implications. The first involves the conceptualization of creativity. The expected hierarchical structure in which domains prevail at the lower end and general creativity constitutes the higher level was confirmed. This supports the viability of a creativity model that has both general and specific components. Our answer to the question of whether there is one creativity or multiple creativities is that both answers are correct, but involve different levels of creativity. The second implication involves the links between values and creativity. Some of these links were predicted based on considerations likely to hold across many cultural contexts (e.g., relations of openness to change with creativity). Understanding other links required careful analysis of specific creative practices in the particular cultural contexts we studied (e.g., relations of Benevolence-Caring values to visual arts creativity in the North Caucasus but not in Central Russia). This implies that the contextualization of creativity is important; a good knowledge of the local context is required to understand the values—creativity nexus.

We measured creativity with self-reports of past behavior. Limitations of recall and self-presentation biases may distort such self-reports. On the other hand, self-reports have the advantage of enabling one to pick up instances of infrequent behavior. Observation or diary methods might be more accurate, but they require closely monitoring participants over extended time periods to capture low frequency creative behavior. Therefore, they are problematic for studying large, representative samples, as done here.

Another limitation is the sampling of domains of creativity. The Dollinger (2003) questionnaire, which yielded only a single general factor (Silvia et al., 2012), included few domains of creativity. By adding more varied items, we were able to distinguish six domains empirically. However, addressing the issue of the domains of creativity more adequately requires a systematic effort to sample the various potential types of creativity in a representative manner. The current research did not assess scientific and literary creativity, for example. Different values may motivate such additional domains.

This study tapped the everyday creativity of ordinary people. Our representative samples included few if any individuals who engaged professionally in creative activities. It is plausible that the values we found to underlie creativity also apply to professionals, but data from samples of professionals are needed to assess that. Such studies would benefit from using the expanded set of values in the refined theory applied here. Finally, our study has demonstrated the need for a contextualized study of creativity. Many studies of creativity have used American samples, often without realizing the cultural constraints of the data obtained in such samples. These studies have yielded many insights in models and assessments, but we clearly need to expand the cultural horizon of our studies of creativity. Paying lip service to the need to study creativity across contexts is not enough.

## Author Contributions

NL initiated the project, coordinated all stages, developed the design of the work and instruments, was involved in data analysis, reporting, and drafting the work. SS was involved in the conceptualization of the study, choice, development of instruments, data analysis, and reporting. FV was involved in the data analysis and reporting. JP was involved in the conceptualization of the study, revised it critically and was involved in drafting the work. EB was involved in the conceptualization, data analysis, and drafting the work.

### Conflict of Interest Statement

The authors declare that the research was conducted in the absence of any commercial or financial relationships that could be construed as a potential conflict of interest.

## Footnotes

^1^In the North Caucasus, Security-Personal values did not inhibit global creativity, though their correlation was negative. They did inhibit creativity in the more formal contexts of work products and machine graphics.

^2^Except Security-Societal.
