# Supplementary material for: Domains of Everyday Creativity and Personal Values
Source: Front Psychol. 2019 Jan 14;9:2681. doi: 10.3389/fpsyg.2018.02681 (PMC6339925; doi:10.3389/fpsyg.2018.02681)
Supplement: Supplementary file 1 [file Table_1.DOCX]

Appendix

Creative Behaviors Questionnaire

I will now read you a list of behaviors that you may have performed in your life, and you, please, tell me, how many times have you done it. Please exclude activities for school or university course work.

| *Card shown to respondents* | | | |
| --- | --- | --- | --- |
| Never did it | Did it once or twice | Did it 3-5 times | Did it more than 5 times |

| 1 | Painted an original picture |
| --- | --- |
| 2 | Made a sculpture |
| 3 | ^A^Made sketches, drafted paintings, graffiti |
| 4 | ^B^Used computer graphics to paint a picture, make collages, web-sites or something else |
| 5 | Made an applied decorative craft piece (from metal, plastic, glass, leather, ceramics, wood, beads, jewelry) |
| 6 | ^A^Made masquerade or festival costumes, designed and made clothing (sewing, knitting crocheting, embroidering) |
| 7 | ^C^Made art photo pictures |
| 8 | ^B^Played a musical instrument in a concert or on the street |
| 9 | ^B^Performed as a singer alone, in an ensemble or a chorus on stage or on the street |
| 10 | ^C^Made a recording (disc) of your own music or of music you performed |
| 11 | ^AC^Composed or wrote the words of a musical piece that was performed |
| 12 | ^AC^Wrote a short story, novel, poem, ballad, play or other piece of literature |
| 13 | ^B^Performed as a dancer alone or as part of an ensemble on stage or on the street |
| 14 | Created or choreographed a dance for performance |
| 15 | Put on a puppet show |
| 16 | ^C^Planned and presented an original speech |
| 17 | Directed a play or other theatrical performance |
| 18 | Participated as an actor in a play or other theater performance or movie (not including crowd scenes),  or as a ‘life sculpture’ |
| 19 | ^A^Drew cartoons or animations on a computer and showed them to other people |
| 20 | Made a movie to show to other people |
| 21 | ^A^Prepared an original floral arrangement, or garden design with plants & flowers |
| 22 | ^B^Developed something new for your work or organization that was adopted (new procedures, rules, organizational arrangements) |
| 23 | ^B^Developed a new product (a machine, computer hardware/software, etc.) |
| 24 | Made an architectural design or plan for a building, house, space/flat, landscape |
| 25 | ^A^Made original posters, placards (including for public meetings) |

^A^Modified from Dollinger et al. (2003); ^B^New item. ^C^Item not used in the final analysis due to sizeable loadings on two or more factors in a preliminary exploratory factor analysis.

Table A Online Supplement

*Correlations between Higher-Order Values and Creativity*

| Value | Central Russia | North Caucasus |
| --- | --- | --- |
|  | Global Creativity | |
| Openness | .22*** | .17*** |
| Self-Enhancement | .00 | .00 |
| Conservation | *-.21**** | *-.15**** |
| Self-Transcendence | .08* | .04 |
|  | Craft | |
| Openness | .14*** | .09** |
| Self-Enhancement | -.09** | -.09** |
| Conservation | *-.12**** | *-.03* |
| Self-Transcendence | *.15**** | *.09*** |
|  | Visual arts | |
| Openness | *.24**** | *.16**** |
| Self-Enhancement | .01 | .02 |
| Conservation | *-.22**** | *-.15**** |
| Self-Transcendence | .05 | .01 |
|  | Performance | |
| Openness | .16*** | .10** |
| Self-Enhancement | -.08* | -.07* |
| Conservation | *-.12**** | *-.05* |
| Self-Transcendence | .10** | .06 |
|  | Theater | |
| Openness | *.23**** | *.12**** |
| Self-Enhancement | -.03 | -.03 |
| Conservation | -.20*** | -.09** |
| Self-Transcendence | .09** | .05 |
|  | Products for Work | |
| Openness | .26*** | .21*** |
| Self-Enhancement | .03 | .04 |
| Conservation | -.24*** | -.21*** |
| Self-Transcendence | .04 | .01 |
|  | Machine Graphics | |
| Openness | *.30**** | *.20**** |
| Self-Enhancement | .05 | .05 |
| Conservation | *-.28**** | *-.21**** |
| Self-Transcendence | .02 | .01 |

*Note.* Correlations printed in italics are significantly different for the two regions in a bootstrapped procedure, using 5000 samples (*p* < .05). **p* < .05. ***p* < .01. ****p* < .001.
